# The *Drosophila* LIN54 homolog Mip120 controls two aspects of oogenesis

**DOI:** 10.1242/bio.025825

**Published:** 2017-05-18

**Authors:** Mei-Hsin Cheng, Laura Andrejka, Paul J. Vorster, Albert Hinman, Joseph S. Lipsick

**Affiliations:** Departments of Pathology, Genetics, and Biology, Stanford University, Stanford, CA 94305-5324, USA

**Keywords:** Mip120, LIN54, Nurse cell, Oogenesis, Germline

## Abstract

The conserved multi-protein MuvB core associates with the Myb oncoproteins and with the RB-E2F-DP tumor suppressor proteins in complexes that regulate cell proliferation, differentiation, and apoptosis. *Drosophila* Mip120, a homolog of LIN54, is a sequence-specific DNA-binding protein within the MuvB core. A mutant of *Drosophila* *mip120* was previously shown to cause female and male sterility. We now show that Mip120 regulates two different aspects of oogenesis. First, in the absence of the Mip120 protein, egg chambers arrest during the transition from stage 7 to 8 with a failure of the normal program of chromosomal dynamics in the ovarian nurse cells. Specifically, the decondensation, disassembly and dispersion of the endoreplicated polytene chromosomes fail to occur without Mip120. The conserved carboxy-terminal DNA-binding and protein-protein interaction domains of Mip120 are necessary but not sufficient for this process. Second, we show that a lack of Mip120 causes a dramatic increase in the expression of *benign gonial cell neoplasm* (*bgcn*), a gene that is normally expressed in only a small number of cells within the ovary including the germline stem cells.

## Abstract (precis)

**Summary:** *Drosophila* Mip120/LIN54, regulates ovarian nurse cell chromosome disassembly and germline-specific gene expression. These functions of Mip120 require its less conserved N-terminus in addition to its CXC DNA-binding and HCH protein-interaction domains.

## INTRODUCTION

**Fig. 1.**
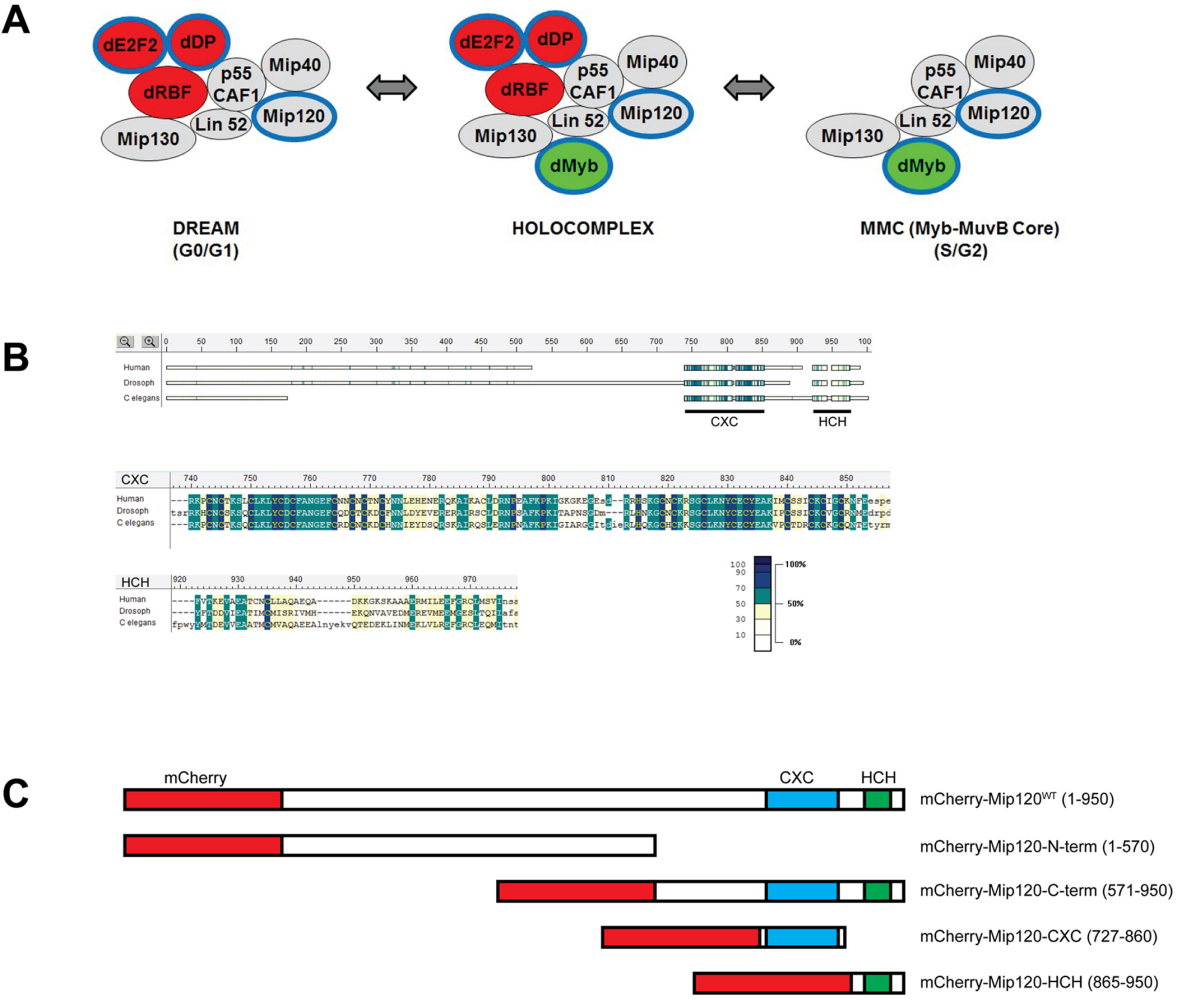
**Mip120 complexes and conserved domains.** (A) The *Drosophila* LIN54 homolog Mip120 participates in three evolutionarily conserved multi-protein complexes. Gray shading indicates members of the MuvB core. Green shading indicates homology with the vertebrate Myb oncogene family. Red shading indicates homology with the vertebrate E2F-DP-RB tumor suppressor axis. Blue outlines indicate sequence-specific DNA-binding proteins. (B) Conservation of the LIN54 and Mip120 proteins in human (NP_919258.2), *Drosophila melanogaster* (NCBI NP_610879.1), and *C. elegans* (NP_502544.1). Thick boxes in the schematic diagram indicate regions with statistically significant homology in a local multi-protein alignment generated with MACAW using the BLOSUM62 scoring matrix (Schuler et al., 1991). Thin bars and gaps indicate unaligned regions. Amino acid sequence alignments are shown for the highly conserved cysteine-rich CXC domain and the helix-coil-helix (HCH) domain (Schmit et al., 2009). Shading reflects the mean-score at each position relative to the entire range of scores in the schematic diagram and in the alignments. (C) Wild-type and mutant *Drosophila* Mip120 proteins used in this study. Red boxes indicate mCherry fluorescent protein tags fused the amino-termini of these Mip120 proteins. Cyan boxes indicate CXC domains. Green boxes indicate HCH domains. Numbering indicates amino acid residues in the *Drosophila* Mip120 A isoform.

Three different avenues of research led to the discovery and initial characterization of the Mip120 and LIN54 family of proteins – biochemistry, genetics in model organisms, and bioinformatics. The Mip120 protein was first identified in *Drosophila* as a component of a multi-protein complex that bound to the origin of DNA replication within a chorion locus that undergoes developmentally programmed gene amplification in ovarian follicle cells during oogenesis (Beall et al., 2002). This complex contained the dMyb oncoprotein, the p55 CAF1 histone chaperone, and three novel Myb-interacting proteins (Mip) that were named based on their relative molecular weights in kilodaltons – Mip130, Mip120, and Mip40 (Fig. 1, MMC). Both dMyb and Mip120 were shown to bind to specific DNA sequences that regulate amplification of the chorion locus. Lin52, a small 18 kDa protein, was later found in this complex. As explained below, the proteins present in this complex, except for dMyb, later became known as the MuvB core, because of their homology to proteins encoded by synMuvB group genes in *C. elegans* (Lipsick, 2004).

A larger holocomplex called Myb-MuvB or *Drosophila* RBF, dE2F2, and dMyb-interacting proteins (dREAM) was then purified from *Drosophila* embryo extracts (Fig. 1, HOLOCOMPLEX) (Korenjak et al., 2004; Lewis et al., 2004). This larger complex contained the heterodimeric dE2F2-dDP DNA-binding proteins and their associated RBF1 or RBF2 tumor suppressor proteins in addition to dMyb and the MuvB core. However, subsequent work showed that the smaller Myb-MuvB core complex (Fig. 1, MMC) and another complex containing only the MuvB core and dE2F2-dDP-RBF1/2 (Fig. 1, DREAM for DP, RB-like, E2F, and MuvB) are more abundant in some cell types, as shown by chromatin immunoprecipitation (ChIP) from the Kc cell line and by immunostaining giant polytene chromosomes in late third instar larval salivary glands (Blanchard et al., 2014; Georlette et al., 2007). These three *Drosophila* complexes containing the MuvB core regulate genes required for progression through the G2 and M phases of the cell division cycle and are also required for proper odorant receptor usage in post-mitotic neurons (Dimova et al., 2003; Georlette et al., 2007; Wen et al., 2008). Since a number of different names and sometimes confusing acronyms have been used for these three complexes in different species, for the sake of clarity we will use only the names shown in Fig. 1 (Lipsick, 2004; Sadasivam and DeCaprio, 2013; van den Heuvel and Dyson, 2008).

Complexes similar to MMC and DREAM were identified in human cell lines (Litovchick et al., 2007; Pilkinton et al., 2007; Schmit et al., 2007). However, the presence of B-MYB/MYBL2 [the functional ortholog of dMyb (Davidson et al., 2005, 2013)] appeared to be mutually exclusive with the presence of E2F4 (a human homolog of dE2F2), DP, and the RB-related proteins p130/RBL2 or p107/RBL1. The large holocomplex can form in human cells when B-MYB is expressed via a heterologous promoter, but presumably the repression of endogenous *B-MYB* gene expression by the DREAM complex normally prevents the co-existence of B-MYB and DREAM (Lam and Watson, 1993; Liu et al., 1996; Marceau et al., 2016; Müller and Engeland, 2010).

In serum-starved human cell lines, the DREAM complex is present during quiescence (G0). Following the addition of serum, Cyclin D levels increase, causing the phosphorylation of retinoblastoma (RB) family proteins and their dissociation from E2F family proteins (Sherr, 2004). The E2F proteins are then subject to degradation by the ubiquitin-proteasome pathway (Hateboer et al., 1996). This in turn relieves transcriptional repression of many S phase-specific genes including *B-MYB*. Synthesis of B-MYB protein results in the appearance of the MMC complex, which activates the transcription of genes required for progression into the G2 and M phases of the cell division cycle. A subsequent switch from B-MYB to FOXM1 has been reported in human cells, but a homologous M phase transcription factor has not yet been identified in *Drosophila* (Sadasivam et al., 2012). Phosphorylation of LIN52 by the DYRK1 protein kinase is required in human cells for reassembly of DREAM in order for cells to enter into quiescence (Litovchick et al., 2011; Tschop et al., 2011). Interestingly, this regulatory phosphorylation site in LIN52 has been conserved in *Drosophila* but not in the nematode worm *C. elegans*.

The second avenue of research that led to the discovery of the Mip120 and LIN54 protein family utilized developmental genetics in nematode worms. All of the components of the biochemically defined DREAM complexes in *Drosophila* and human turned out to be encoded by homologs of the genetically defined synMuvB complex in *C. elegans* (Lipsick, 2004). In brief, the abnormal presence of multiple birth canals in certain worm mutants was designated as the multivulval (Muv) phenotype (Sternberg and Han, 1998). Dominant gain-of-function mutations of a highly conserved receptor tyrosine kinase signaling pathway including the homologs of *EGF* and the *EGFR*, *RAS*, *RAF*, and *ETS* proto-oncogenes were shown to cause this Muv phenotype. Conversely, loss-of-function mutants of the same genes caused a vulvaless (Vul) phenotype.

One unusual Muv strain of worms instead contained two recessive loss-of-function mutants (*lin-8* and *lin-9*) that were both required for this synthetic mutant phenotype (Horvitz and Sulston, 1980). Further genetic screens revealed two groups of mutants such that any loss-of-function mutant from group A (synMuvA) could cooperate with any loss-of-function mutant from the group B (synMuvB) to cause a Muv phenotype (Ferguson and Horvitz, 1989). These results implied that these two groups of genes were required redundantly to repress the RAS pathway. The synMuvB group included homologs of RB, E2F, DP, and all the other members of the DREAM complex (Ceol et al., 2006). In nematodes this complex is also required for the repression of germline genes in somatic cells and for normal levels of X chromosome gene expression (Petrella et al., 2011; Tabuchi et al., 2014). Some of the human homologs of this complex were named for their nematode counterparts rather than their *Drosophila* counterparts (LIN9=Mip130, LIN54=Mip120, LIN37=Mip40, LIN52), whereas others had pre-existing names (p107/RBL1, p130/RBL2, E2F4, DP, RBBP4 or RBAP48=p55CAF1). Remarkably, *C. elegans* does not contain an ortholog of *Drosophila* dMyb and human B-MYB, although other more distantly related metazoans do, including sea anemone and hydra (Andrejka et al., 2011; Davidson et al., 2004, 2005, 2013).

A third avenue of research that led to Mip120 and LIN54 family of proteins was a bioinformatic analysis of transcriptional regulatory elements of cell cycle-regulated genes in vertebrate cell lines. Two distinct *cis*-acting motifs, the cell cycle-dependent element (CDE) and the cell cycle genes homology region (CHR), were identified in the promoters of many of these genes (Müller and Engeland, 2010). Some cell cycle-regulated genes contain both of these sites, separated by only four base pairs. Other cell cycle-regulated genes contain only a CHR. Although the CDE is not a canonical E2F binding site (TTTCCCGC), a variety of experimental approaches showed that E2F-DP heterodimers can bind to CDE motifs both *in vitro* and *in vivo*. The identity of the CHR-binding protein remained a mystery until a bacterially expressed CXC domain containing two tandem highly conserved cysteine-rich regions near the C-terminus of human LIN54 was shown to bind specifically to a CHR motif (TTTGAA) within the human *cdc2* promoter (Fig. 1) (Schmit et al., 2009). Furthermore, an adjacent helix-coil-helix (HCH) domain at the extreme C-terminus of LIN54 was shown to be necessary and sufficient for co-precipitation of human B-MYB and p130/RBL2 proteins in pull-down assays *in vitro* (Schmit et al., 2009). These results were corroborated by studies in murine cell lines using SILAC and DNA affinity chromatography of nuclear extracts showing that the DREAM complex bound to adjacent CDE/CHR motifs (Müller et al., 2012). Furthermore, the CHR was shown to be essential for DREAM binding. Recently, the *Drosophila* Mip120 protein was shown to interact with the L(3)MBT chromatin-binding tumor suppressor protein *in vitro* and to be required for its localization to chromosomes *in vivo* (Blanchard et al., 2014).

The structure of the human LIN54 DNA-binding domain was recently solved by X-ray crystallography (Marceau et al., 2016). The two tandem cysteine-rich regions of LIN54 that constitute the CXC domain bind to DNA in a novel manner with two tyrosine residues inserted into the minor groove of the double helix. This minor groove binding provided an explanation for the very close spacing between adjacent CDE and CHR sites, since E2F-DP heterodimers bind to their recognition motifs in the major groove. Molecular modeling studies suggest that simultaneous occupation of a combined CDE-CHR site by E2F-DP and LIN54 is indeed possible.

A null mutant of *Drosophila mip120* was previously created by imprecise P element excision (Beall et al., 2007). Homozygous mutant flies were reported to have a range of phenotypes including reduced longevity, adult eye defects, male sterility, and female sterility. *Drosophila* oogenesis has been intensively studied and provides a powerful system for a genetic analysis of the development of the germline and its interactions with supporting somatic cells (Fuller and Spradling, 2007; Gilboa and Lehmann, 2004; Spradling, 1993). Furthermore, a variety of different types of cell cycles are used in this complex process (Calvi and Spradling, 1999; Lee and Orr-Weaver, 2003; Lilly and Duronio, 2005). We have therefore investigated the role of Mip120 in *Drosophila* oogenesis.

## RESULTS

### Loss of Mip120 causes an arrest of oogenesis during the transition between stages 7 and 8

We began our investigation of the role of Mip120 in oogenesis by obtaining the only reported mutant allele of *mip120*, which was kindly provided by the laboratory of Michael Botchan (University of California Berkeley). The *mip120*^67^ null allele was previously generated by imprecise P-element excision that deleted the first three exons of *mip120* and the essential *EfTuM* gene that lies within the first intron of *mip120* (Fig. S1) (Beall et al., 2007). To rescue the deleted *EfTuM* gene, a transgene that contained the entire first intron of *mip120* was recombined onto the same chromosome as *mip120^67^* to generate *w*; *mip120*^67-9a-9^/CyO flies. Although we were able to confirm the presence of the *mip120*^67^ deletion, homozygous mutant females were no longer sterile. We also did not observe any of the previously described eye phenotypes. Since mutations that reduce fertility have a selective disadvantage, we reasoned that continual passage of the stocks had resulted in the accumulation of genetic or epigenetic modifiers that compensated for the absence of Mip120. We therefore tested for female sterility in flies that were hemizygous for *mip120^67-9a-9^* over *Df(2R)BSC274*, a chromosomal deficiency that contains a 162.8 kb deletion within the second chromosome that removes 31 genes including *mip120* (Cook et al., 2012). The *mip120^67-9a-9^*/*Df(2R)BSC274* females were completely sterile (Table 1); however, we did not observe any of the previously reported eye phenotypes.

**Table 1.**
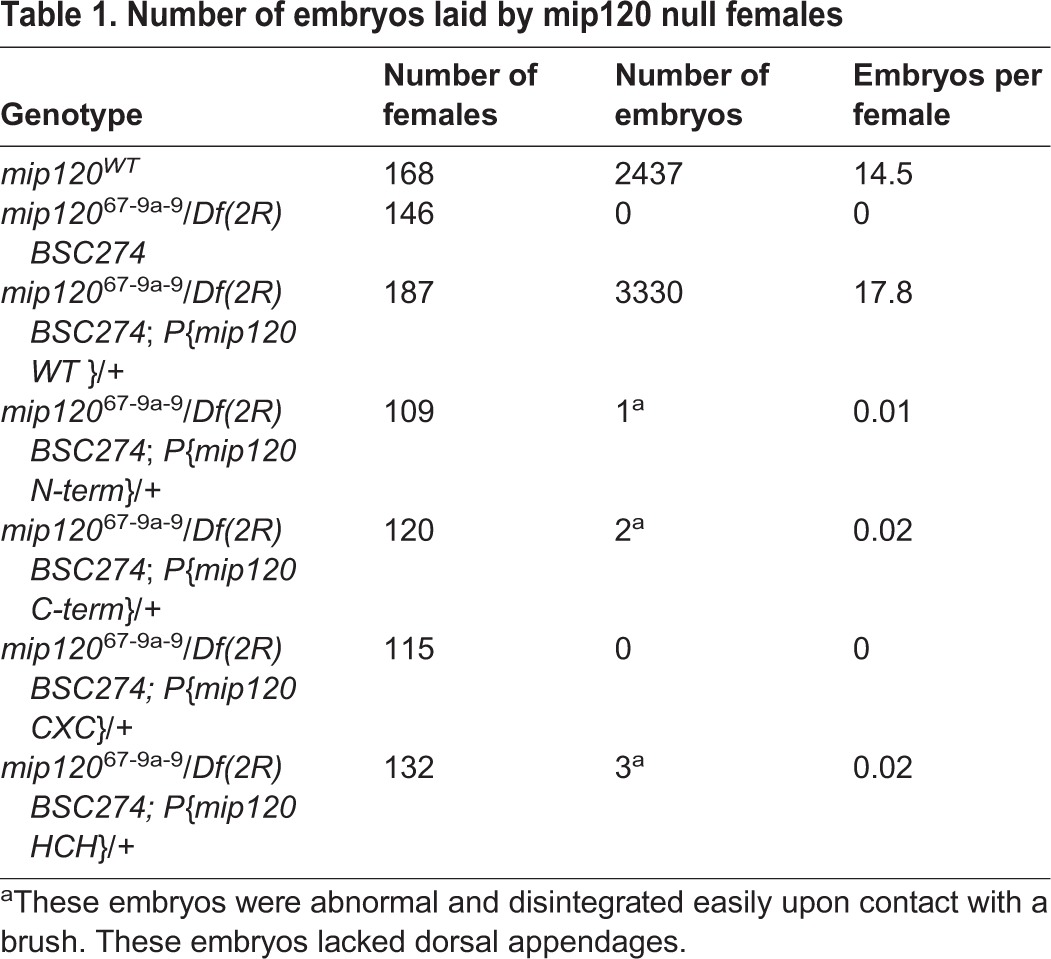
**Number of embryos laid by mip120 null females**

**Fig. 2.**
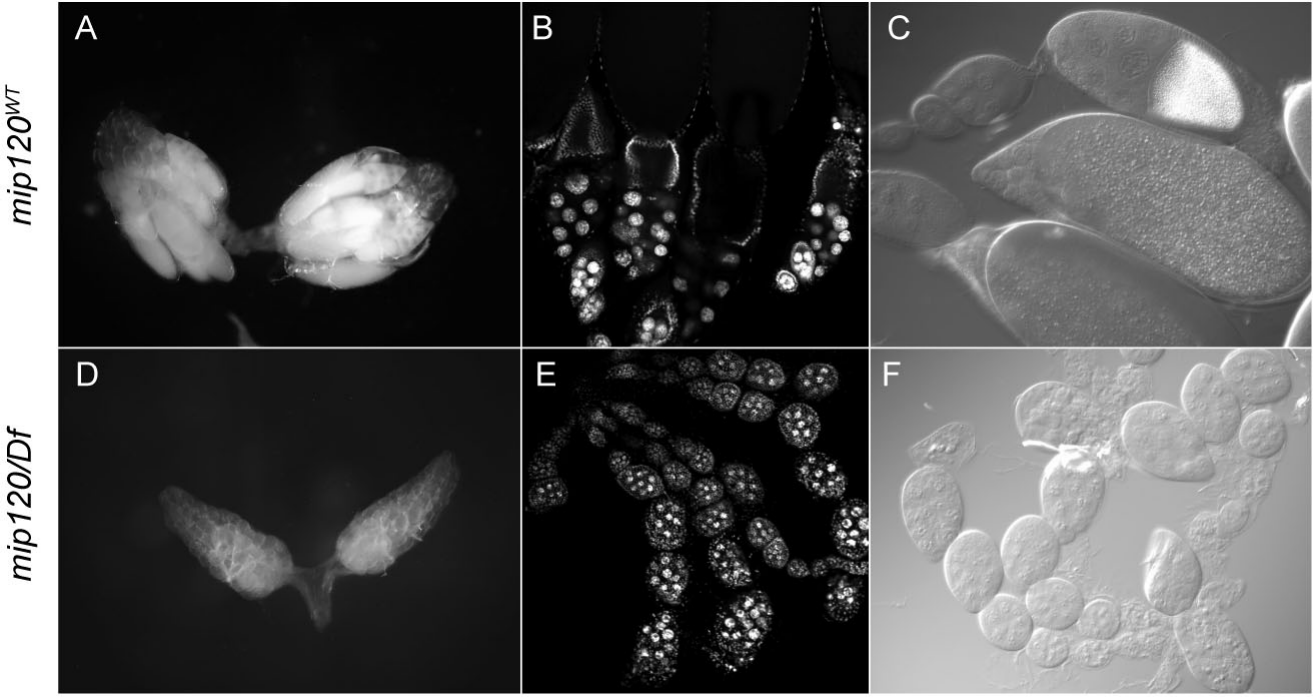
***mip120* null mutants have abnormal egg chamber development.** Ovaries and egg chambers were isolated from control flies (*mip120^WT^*) (A-C) and from mutant *mip120^67-9a-9^*/*Df(2R)BSC274* flies (*mip120*/*Df*) (D-F). (A,D) Bright-field images of freshly dissected whole ovaries from adult females. (B,E) Confocal images of fixed egg chambers stained with TO-PRO-3. (C,F) Nomarski interference contrast images of fixed egg chambers.

**Fig. 3.**
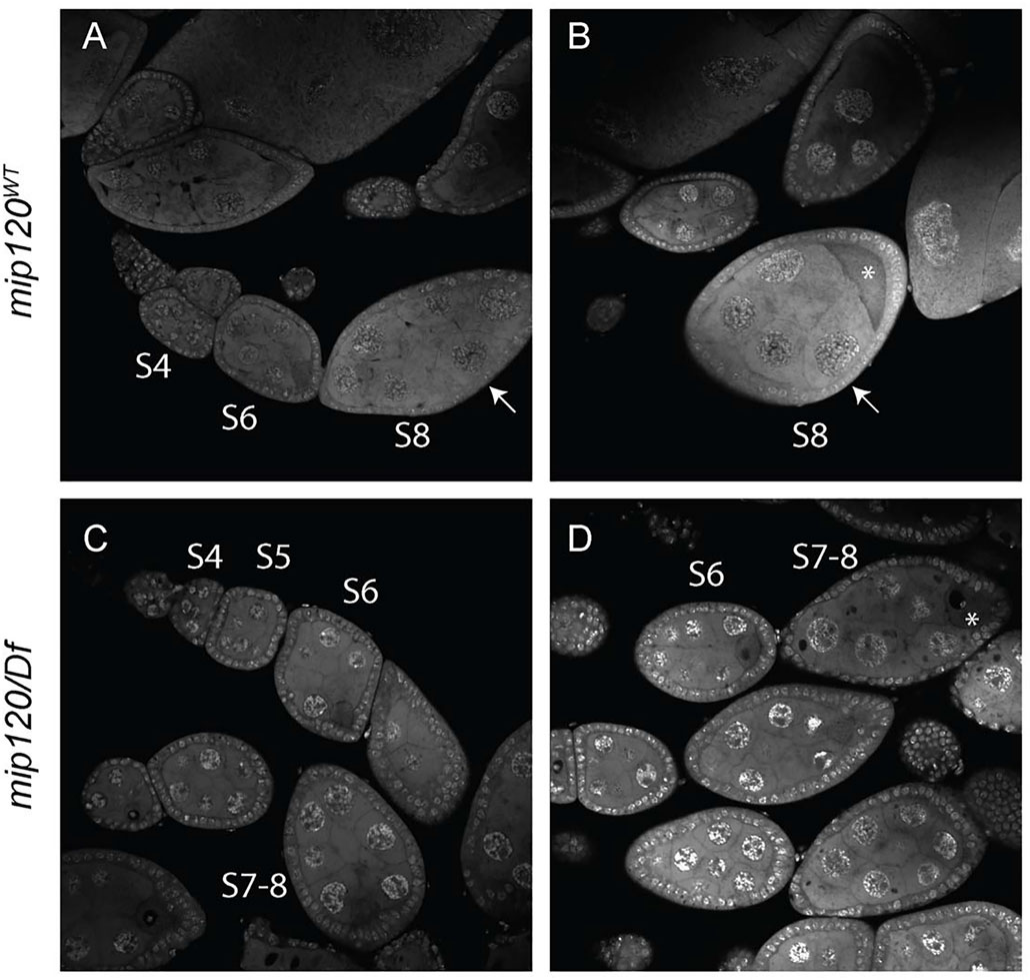
***mip120* null egg chambers arrest during the transition between stages 7 and 8.** Egg chambers from wild-type and *mip120^LL07629^ /Df* ovaries were fixed, stained with TO-PRO-3, and washed briefly in PBS to permit visualization of both nuclear DNA and cytoplasmic RNA. (A,B) The two confocal images of wild-type egg chambers are of the same specimen taken at different planes in the z-axis and translated along the x-axis in order to facilitate egg chamber staging. The arrows point to the same egg chamber for reference. (C,D) The two confocal images of mutant egg chambers are of different specimens chosen to facilitate staging and to display the uniformity of the terminal phenotype, respectively. S4 indicates stage 4, S5 indicates stage 5, and so on. The asterisks indicate oocytes.

**Fig. 4.**
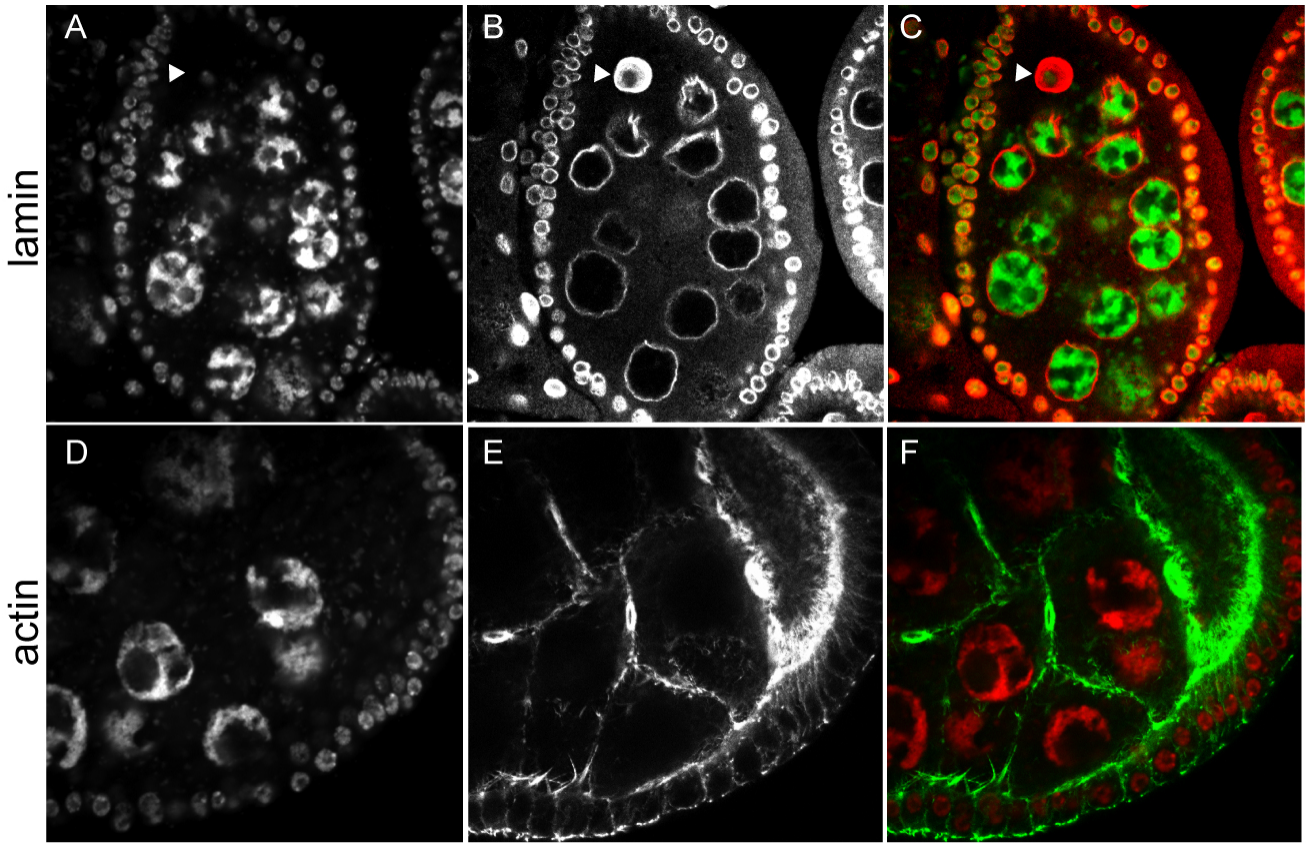
***mip120* null egg chambers have a single oocyte.** Stage 7-8 egg chambers from *mip120^67-9a-9^/Df* mutant females were fixed, stained with TO-PRO-3 (A,D) and with either anti-lamin antibody to mark the nuclear envelope (B) or phalloidin to mark the actin cytoskeleton (E), then imaged by confocal microscopy. In the merged images (C,F), TO-PRO-3 is shown in green and either lamin or actin in red. The arrowhead indicates an oocyte nucleus (A-C).

Ovaries dissected from *mip120^67-9a-9^*/*Df(2R)BSC274* females were much smaller than those from control females (Fig. 2). Egg chamber development showed a consistent arrest in all ovarioles with a terminal egg chamber phenotype that appeared to be in transition between stages 7 and 8 (King, 1970; Spradling, 1993). Furthermore, the ovaries of females with a new independent mutant allele *mip120^LL07629^* described in detail below displayed the same phenotype (Fig. 3). The most mature egg chambers in each mutant ovariole were ovoid and contained a uniform layer of cuboidal follicle cells surrounding fifteen nurse cells and a posterior oocyte. The ploidy of these nurse cells as estimated by nuclear diameter appeared similar to that of the anterior nurse cells in normal stage 8 egg chambers (256C). However, the mutant posterior nurse cells had not undergone an additional round of endoreplication, resulting in the increased ploidy (512C) normally seen in posterior nurse cells at stage 8. Apparently normal actin-rich ring canals connected the nurse cells and the posterior oocyte (Fig. 4). The oocyte had a distinct lamin-rich germinal vesicle containing a small karyosome with decondensed DNA. However, the oocyte did not appear to have begun to accumulate yolk as is typical of stage 8 egg chambers. This absence of yolk was assessed by Nomaski interference microscopy, autofluorescence, and decreased staining of cytoplasmic RNA.

### Loss of Mip120 causes persistent chromosomal condensation and failure of chromosome disassembly and dispersion in ovarian nurse cells

**Fig. 5.**
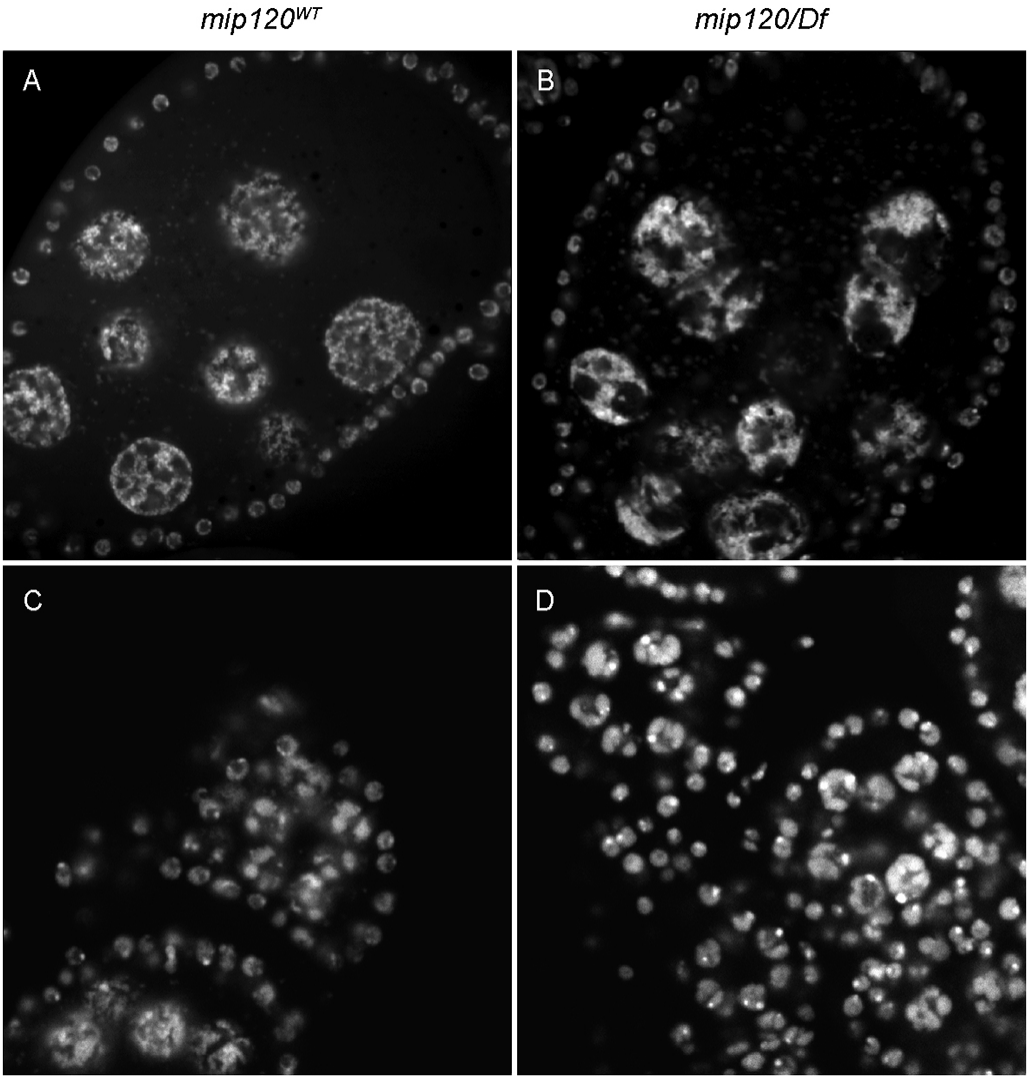
***mip120* null egg chambers have a condensed nurse cell DNA phenotype.** Stage 7-8 egg chambers from *mip120^WT^* control (A) and *mip120^67-9a-9^/Df* mutant (B) females were fixed, stained with TO-PRO-3, and imaged by confocal microscopy. Stage 2-5 egg chambers from *mip120^WT^* control (C) and *mip120^67-9a-9^/Df* mutant (D) females were fixed, stained, and imaged by confocal microscopy.

Ovarian nurse cells normally display a series of distinct chromosomal morphologies and changes in ploidy during oogenesis (Dej and Spradling, 1999; Hammond and Laird, 1985). The first four rounds of endoreplication create visibly banded polytene chromosomes with pairing of the homologs. After each S phase, these chromosomes condense into blob-like nuclear structures that are visible through stage 5 of oogenesis. After the fifth endocycle, each 64C chromosome undergoes a mitosis-like disassembly into 32 distinct pairs of chromatids that disperse throughout the nucleus by stage 6 of oogenesis. This dispersed state is retained during subsequent rounds of endoreplication and persists through the rest of oogenesis. Nurse cells in *mip120^67-9a-9^*/*Df(2R)BSC274* egg chambers appeared normal through stage 5, but thereafter the chromosomes remained condensed with an aberrant nurse cell morphology (Fig. 5). At the point of arrest during the transition between stages 7 and 8, the chromosomes appeared not to have undergone the mitosis-like disassembly and dispersion that normally occur during stage 5.

**Fig. 6.**
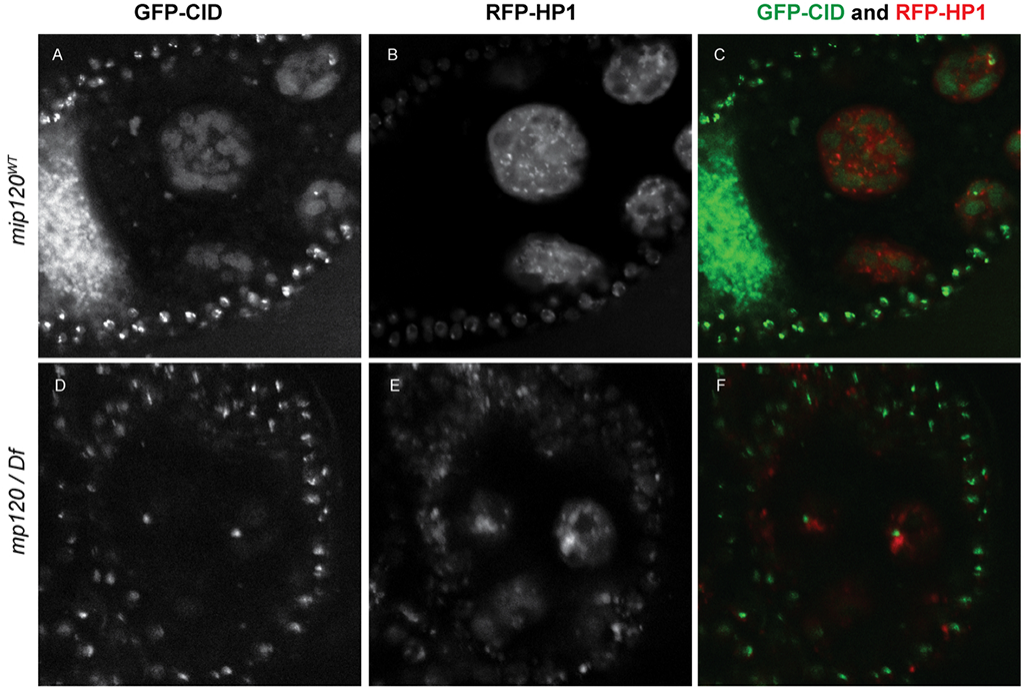
**Failure of chromosome disassembly in *mip120* null ovarian nurse cells.** Stage 7-8 egg chambers from *mip120^WT^* control (A-C) and *mip120^67-9a-9^/Df* mutant (D-F) females were dissected, mounted in halocarbon oil and imaged live by confocal microscopy. Both genotypes also contained transgenes expressing GFP-CID and RFP-HP1. Single channel images of GFP-CID (A,D) and RFP-HP1 (B,E) are shown, as are merged images with GFP-CID (green) and RFP-HP1 (red) (C,F). Autofluorescent yolk material appears in the GFP channel in the *mip120^WT^* egg chamber. An outer layer of small follicle cell nuclei surround the large interior nurse cell nuclei.

To further investigate whether disassembly and dispersion of the polytene chromosomes failed to occur in nurse cells in the absence of Mip120, we introduced transgenes expressing RFP fused to heterochromatin protein 1 (RFP-HP1) and GFP fused to the centromeric CENPA family histone H3 variant CID (GFP-CID), each under control of its native promoter, into either wild-type *mip120* control egg chambers or into *mip120^67-9a-9^*/*Df(2R)BSC274* mutant egg chambers. To avoid artifacts of fixation, these egg chambers were visualized by live confocal microscopy immediately after dissection (Fig. 6). Nurse cells of control stage 7 and 8 egg chambers with wild-type *mip120* contained numerous small distinct chromosomes, each with discrete HP1-positive heterochromatic foci and an unexpectedly diffuse incorporation of CID. In contrast, nurse cells of *mip120^67-9a-9^*/*Df(2R)BSC274* egg chambers arrested during the transition from stage 7 to 8 contained large condensed chromosomes, each with a single discrete CID-positive centromere. Adjacent to each CID-positive centromere was a region of densely HP1-positive pericentric heterochromatin. These results demonstrate that the large condensed chromosomes in the mutant nurse cells have indeed failed to undergo chromosome disassembly and dispersion.

**Fig. 7.**
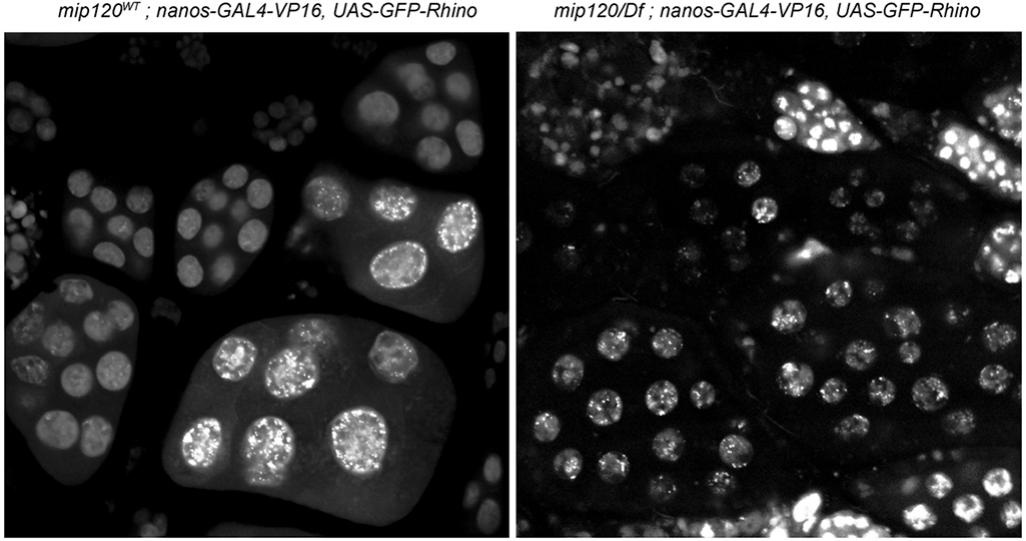
**Ectopic expression of Rhino does not rescue *mip120* null ovarian nurse cells.** Ovarioles from *mip120^WT^* control (left) and *mip120^67-9a-9^/Df* mutant (right) females were dissected, mounted in halocarbon oil and imaged live by confocal microscopy. Both genotypes also contained a *nanos-GAL4-VP16* transgene driving germline expression of a *UAS-GFP-Rhino* transgene. Images show GFP-Rhino present within the nuclei of egg chambers of both genotypes. However, no egg chambers beyond stage 8 are seen in the *mip120/Df* ovarioles.

The Rhino protein is a member of the HP1 chromodomain family that is present only in females, predominantly in the germline. Mutants of *rhino* have been reported to cause a persistence of the blob-like morphology of nurse cell nuclei, although egg chambers continue to develop well beyond stage 8 through egg deposition (Volpe et al., 2001). We therefore introduced a UAS-GFP-Rhino transgene under control of the nanos-GAL4-VP16 germline driver into *mip120^67-9a-9^*/*Df(2R)BSC274* females. This transgene combination has previously been shown to rescue the *rhino* null mutant phenotype (Klattenhoff et al., 2009). To again avoid artifacts of fixation, these egg chambers were visualized by live confocal microscopy immediately after dissection (Fig. 7). The GFP-Rhino protein was clearly expressed in the *mip120^67-9a-9^*/*Df(2R)BSC274* mutant egg chambers and decorated the chromosomes of nurse cell nuclei. However, neither the persistent undispersed chromosome condensation phenotype nor the arrest of oogenesis during the transition between stages 7 and 8 were rescued by ectopic expression of GFP-Rhino. Furthermore, the GFP-Rhino protein was present in punctate structures in the nurse cell nuclei of stage 7-8 *mip120* mutant egg chambers but not the stage 7-8 control egg chambers, as might be expected for chromosomes that have not undergone normal disassembly after stage 5.

### The Mip120 DNA-binding domain is necessary but not sufficient for rescue of oogenesis

**Fig. 8.**
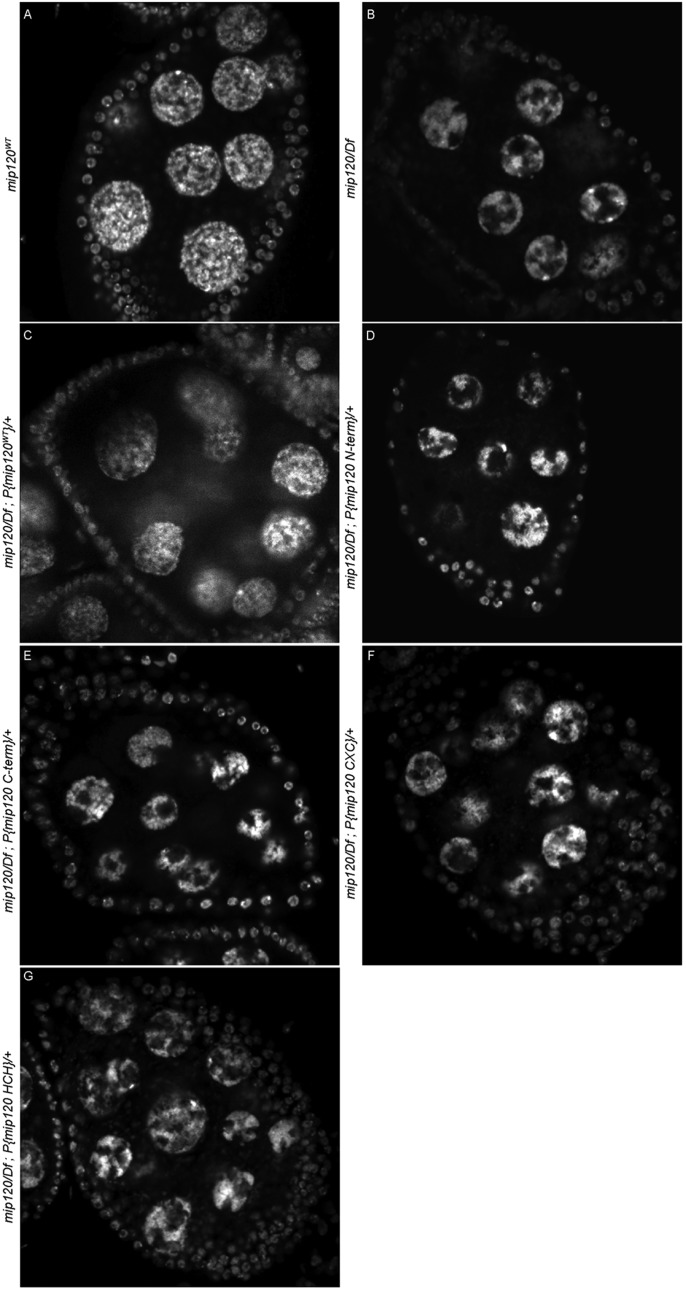
**The less conserved N-terminus is required along with the CXC and HCH domains for Mip120 to rescue the *mip120* null condensed nurse cell DNA phenotype.** Fixed egg chambers from *mip120^WT^* (A), *mip120^67-9a-9^/Df* (B), *mip120^67-9a-9^/Df*; *P{mip120}/*+ (C), *mip120^67-9a-9^/Df*; *P{mip120 N-term}*/+ (D), *mip120^67-9a-9^/Df*; *P{mip120 C-term}*/+ (E), *mip120^67-9a-9^/Df*; *P{mip120 CXC}*/+ (F), and *mip120^67-9a-9^/Df*; *P{mip120 HCH}*/+ (G) females were stained with TO-PRO-3 and imaged by confocal microscopy.

We wished to determine whether this unusual nurse cell nuclear phenotype was indeed due to the mutant allele of *mip120*, rather than being due to potential background mutations. We therefore constructed a rescue P element that expressed a wild-type Mip120 protein fused in-frame at its N-terminus to the mCherry fluorescent protein under the control of a *mip120* promoter. This transgene efficiently rescued the nurse cell nuclear phenotype, abolished the arrest of oogenesis, and restored female fertility in *mip120^67-9a-9^*/*Df(2R)BSC274* females (Fig. 8; Tables 1 and 2).

**Table 2.**
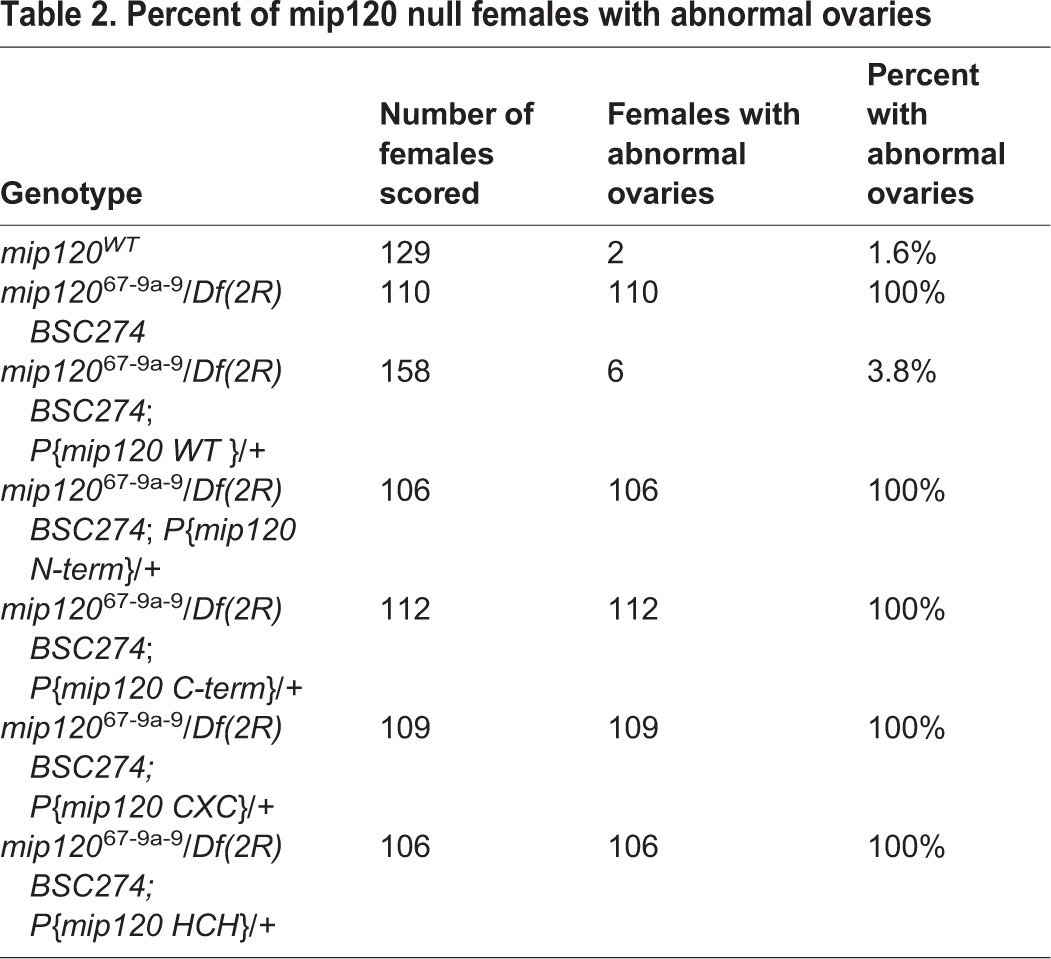
**Percent of mip120 null females with abnormal ovaries**

**Fig. 9.**
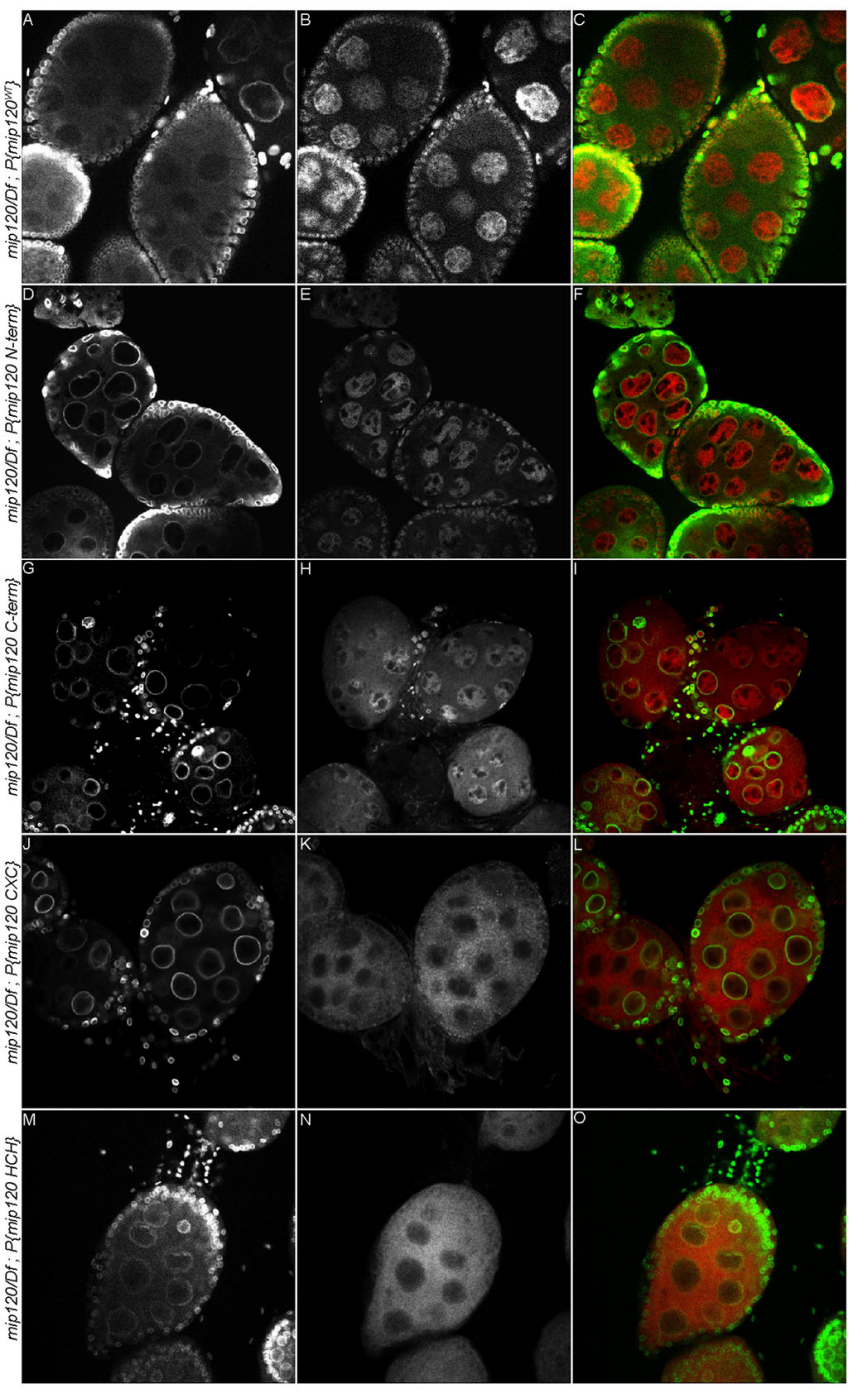
**Mip120 CXC and HCH domains are not sufficient for nuclear localization in *mip120* null egg chambers.** Fixed egg chambers from *mip120^67-9a-9^/Df*; *P{mip120}/*+ (A-C), *mip120^67-9a-9^/Df*; *P{mip120 N-term}*/+ (D-F), *mip120^67-9a-9^/Df*; *P{mip120 C-term}*/+ (G-I), *mip120^67-9a-9^/Df*; *P{mip120 CXC}*/+ (J-L), and *mip120^67-9a-9^/Df*; *P{mip120 HCH}*/+ (M-O) females were fixed and immunostained with anti-Lamin (A,D,G,J,M) to detect the nuclear envelope and anti-Cherry fluorescent protein (B,E,H,K,N) to detect mCherry-Mip120 fusion proteins, then imaged by confocal microscopy. Merged images (C,F,I,L,O) show Lamin in green and Cherry in red.

We had previously shown that the highly conserved DNA-binding domain of Myb was not required to rescue many aspects of the *Myb* null mutant phenotype (Andrejka et al., 2011; Wen et al., 2008). We therefore wished to test whether the DNA-binding domain of Mip120, the only member of the MuvB core currently known to bind directly to DNA, was required for oogenesis. For this purpose, we constructed and tested four additional P elements that encoded deletion mutants of Mip120 fused in-frame at their N-termini to the mCherry fluorescent protein under control of a *mip120* promoter. These four mutants included the N-terminus lacking the CXC and HCH domains, the C-terminus containing the CXC and HCH domain but lacking the less conserved N-terminus, the CXC DNA-binding domain alone, or the HCH protein-protein interaction domain alone (Fig. 1). Unlike the wild-type protein, none of these mutant proteins were able to rescue the nurse cell nuclear phenotype, the arrest of oogenesis, or female fertility in *mip120^67-9a-9^*/*Df(2R)BSC274* females (Fig. 8, Tables 1 and 2). However, all of the mCherry-Mip120 proteins were expressed in the ovaries of *mip120^67-9a-9^*/*Df(2R)BSC274* females containing these transgenes (Fig. 9). Interestingly, the N-terminus, which lacked the CXC and HCH domains, still localized to the nucleus. The C-terminus, which contained the CXC and HCH domains, was both nuclear and cytoplasmic. However, neither the CXC nor the HCH domain alone were sufficient for nuclear localization.

### Absence of Mip120 in follicle cells does not affect nurse cell morphology

Complex patterns of reciprocal signaling between germline and somatic cells occur throughout *Drosophila* oogenesis and early embryogenesis (Deng and Lin, 2001; Roth and Lynch, 2009; Schupbach, 2016). Therefore, we wished to determine whether the unusual nurse cell morphology caused by the absence of Mip120 required the activity of this protein in the surrounding follicle cell epithelium. As described above, genetic analysis of the *mip120^67^* allele is complicated by the need to rescue the essential *EfTuM* gene within the first intron of *mip120* that is also removed by this deletion mutant (Beall et al., 2007). To simplify mosaic analysis, we sought a new *mip120* mutant that did not affect *EfTuM.* Using the FlyBase resource (http://flybase.org/), we identified several previously described lethal or semi-lethal transposon insertions in or near the locus and tested them for rescue by our *P{mCherry-Mip120^WT^}* transgene. A piggyback transposon insertion *PBac{SAstopDsRed}LL07629* near the 5′ end of the second exon of *mip120* was successfully rescued by *P{mCherry-Mip120^WT^}* in the absence of an *EfTuM* transgene (Fig. S1, Table S1). Henceforth, this new mutant allele will be referred to as *mip120^LL07629^*.

**Fig. 10.**
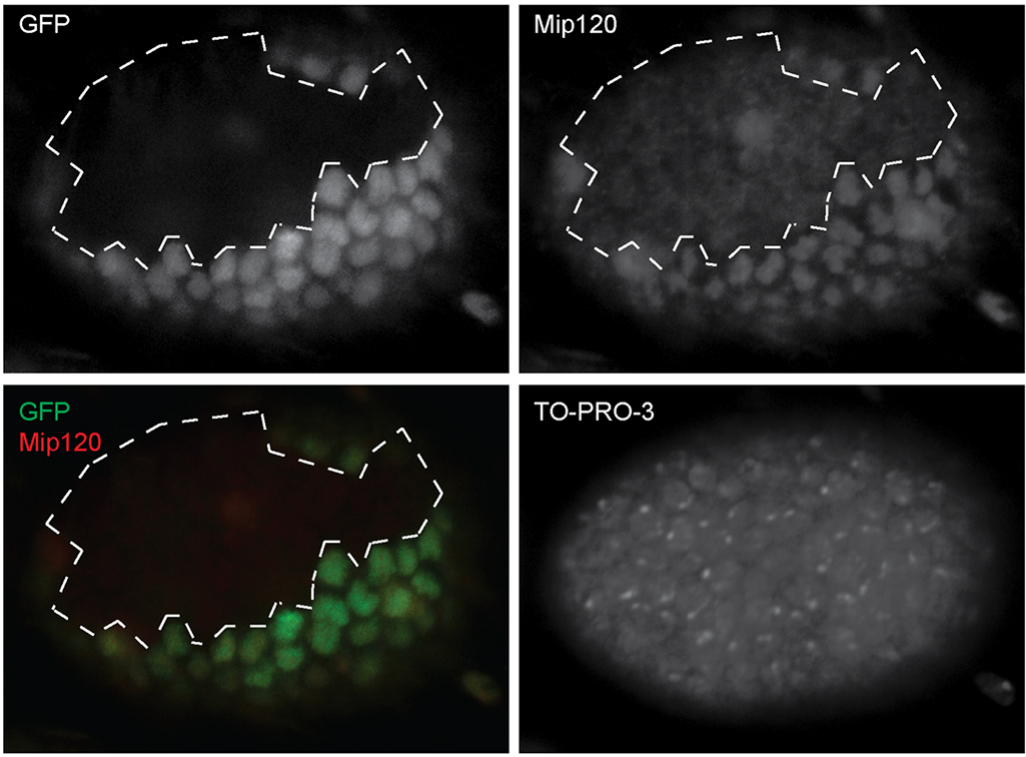
**A mutant clone homozygous for *mip120^LL07629^* has greatly diminished Mip120 protein levels.** *hsFLP/+;* FRT42B, *mip120^LL07629^/FRT42B, Ubi-GFP-nls* females were heat-shocked to induce the site-specific FLP recombinase, generating homozygous GFP-negative clones that were also homozygous for the mutant allele, *mip120^LL07629^*. Egg chambers were dissected, fixed, then stained with anti-Mip120 antibodies and TO-PRO-3. Follicle cells on the surface of the egg chamber were imaged by confocal microscopy for: GFP (top left); anti-Mip120 (top right); GFP (green) and anti-Mip120 (red) (bottom left); and TO-PRO-3 (bottom right). The boundary of the GFP-negative mutant clone is indicated by a dashed line. The TO-PRO-3 bright dots within the nuclei represent heterochromatin-dense chromocenters.

**Fig. 11.**
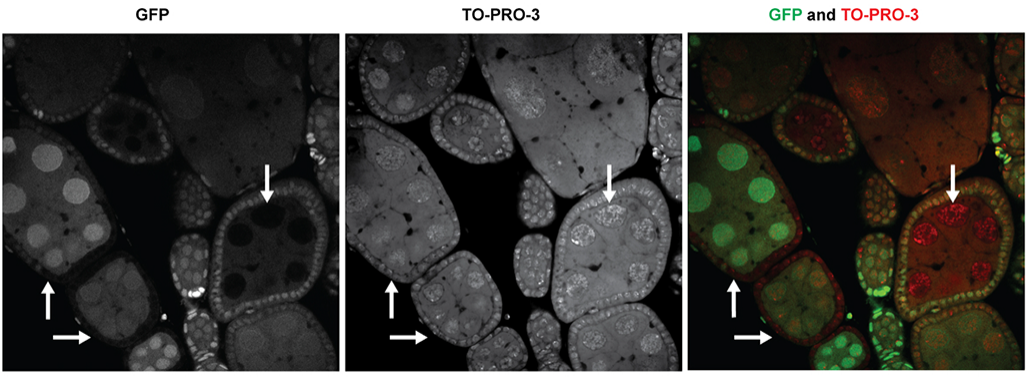
**Mip120 is not required in adjacent follicle cells for normal nurse cell morphology.** Ovaries were dissected from heat-shocked *hsFLP/+; FRT42B, mip120^LL07629^/FRT42B, Ubi-GFP-nls* females as described in Fig. 9, then egg chambers were fixed, dissected, stained with TO-PRO-3 and imaged by confocal microscopy for: GFP (left); TO-PRO-3 (middle); or GFP (green) together with TO-PRO-3 (red) (right). Rightward and upward arrows indicate two egg chambers with large patches of GFP-negative homozygous *mip120^LL07629^* mutant follicle cells adjacent to GFP-positive heterozygous *mip120^WT^/mip120^LL07629^* nurse cells. Downward arrows indicate an egg chamber with GFP-negative homozygous *mip120^LL07629^* mutant nurse cells surrounded by GFP-positive heterozygous *mip120^WT^/mip120^LL07629^* follicle cells.

We then used a heat shock-inducible version of the FLP-FRT site-specific recombination system to generate homozygous *mip120^LL07629^/mip120^LL07629^* mutant clones of cells in *mip120^LL07629^/mip120^WT^* females (Golic, 1991). We used a nuclear localizing nlsGFP expressed under the control of the *ubiquitin* promoter as a dominant marker for the presence of the wild-type *mip120* allele in both germline and somatic cells of the ovary (Luschnig et al., 2004). As expected, immunostaining with anti-Mip120 antibodies revealed a substantial reduction of the Mip120 protein in homozygous mutant cells marked by the absence of nlsGFP (Fig. 10). Furthermore, we were able to identify egg chambers in which some or all of the follicle cells lacked nlsGFP, and therefore also lacked Mip120 (Fig. 11). However, in the absence of Mip120 in the follicle cells, the adjacent *mip120^LL07629^/mip120^WT^* nurse cells displayed a normal nuclear morphology with chromosome disassembly and dispersion. Conversely, egg chambers in which germline cells lacking Mip120 were surrounded by *mip120^LL07629^/mip120^WT^* follicle cells displayed an abnormal nurse cell nuclear morphology. These results imply a cell-autonomous role for Mip120 in nurse cells.

### Abnormally high levels of *bgcn* expression in the absence of Mip120

The multi-protein complexes containing Mip120 have previously been reported to bind to 32% of all promoters in the *Drosophila* Kc cell line (Georlette et al., 2007). In an attempt to identify genes that might be directly deregulated by the absence of Mip120 during oogenesis, we analyzed the publicly available chromatin immunoprecipitation data from that study. Specifically, we searched for genes whose promoters were occupied by the generally repressive Mip120 protein but that were not occupied by the activator Myb. We then used the DAVID resource version 6.7 (https://david.ncifcrf.gov/home.jsp) to narrow this set of 234 genes to those annotated with the GO terms ‘gamete generation’ or ‘oogenesis’. The expression levels of the resulting five genes (*bgcn*, *chic*, *dap*, *dia*, *egg*) were then measured by qPCR in ovaries dissected from *mip120^67-9a-9^*/*Df(2R)BSC274* mutants or from *mip120^WT^* controls.

**Fig. 12.**
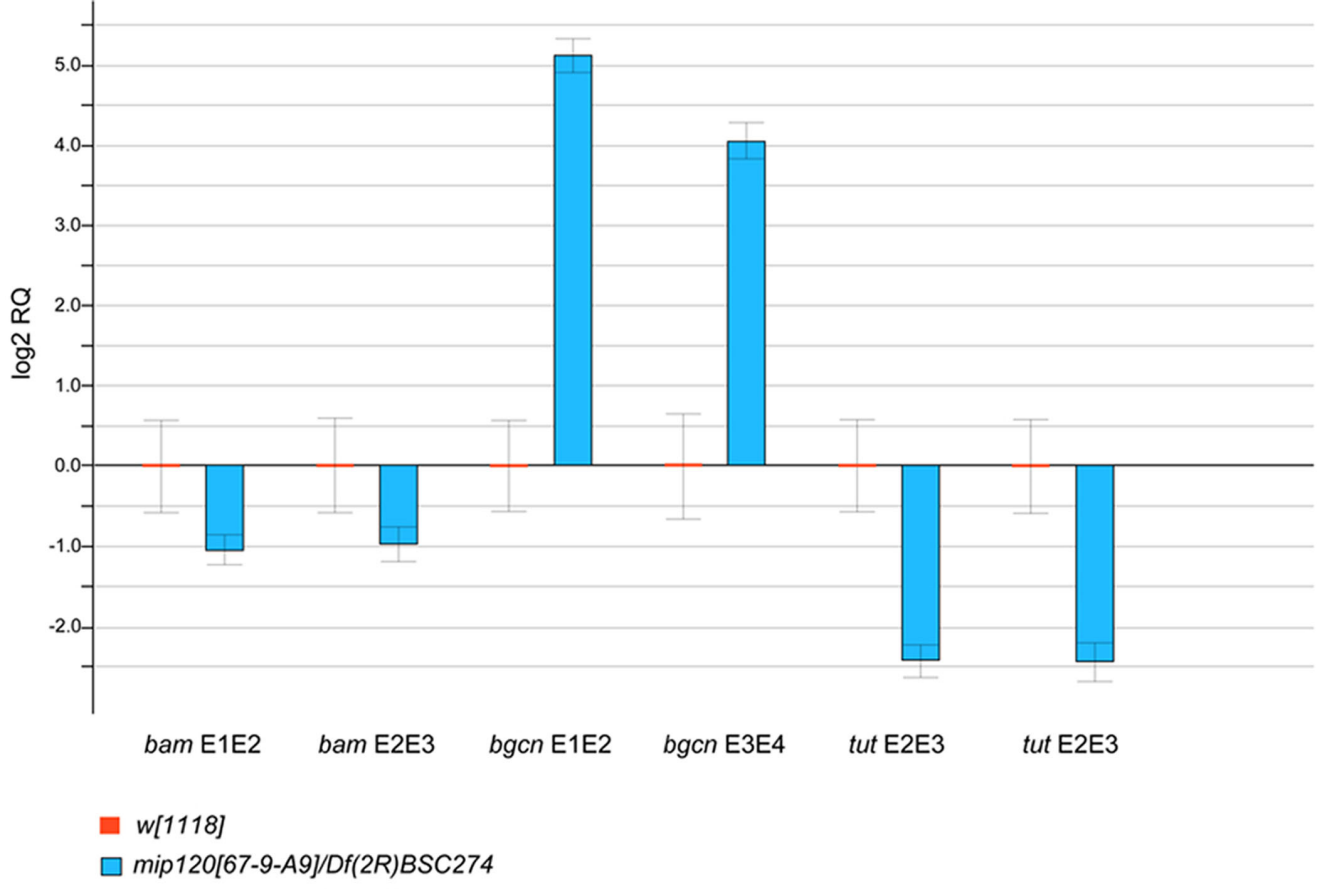
**Levels of *bgcn* RNA are greatly increased in *mip120* null ovaries.** Ovaries from *mip120^WT^* control (*w^1118^*) and *mip120^67-9a-9^*/*Df(2R)BSC274* mutant females were dissected and qPCR was used to quantitate RNA levels of *bam*, *bgcn*, and *tut*. Two different sets of PCR primers flanking an intron were used for each gene, with the relevant exons indicated as E1, E2, E3, E4. Errors bars indicate RQ min and RQ max values for three technical replicates.

Among these five genes, only *bgcn* showed a dramatic alteration with a greater than tenfold increase in RNA levels (Fig. S2). Since the Bgcn protein acts in a complex together with the Bam (bag of marbles) and Tut (tumorous testis) proteins to regulate translation of germline genes during differentiation, we also measured expression levels of these two additional genes (Fig. 12) (Chen et al., 2014; Li et al., 2009). In contrast to the large increase in *bgcn* RNA levels in the absence of Mip120, *bam* and *tut* showed moderately decreased RNA levels.

Several known targets of negative regulation by Mip120 and positive regulation by Myb were also tested (*aurA*, *aurB*, *polo*). As expected, their expression was moderately increased in the absence of Mip120 (DeBruhl et al., 2013; Georlette et al., 2007; Wen et al., 2008) (Fig. S2). Since condensins have previously been shown to regulate nurse cell chromosomal and nuclear architecture, we also measured RNA levels of *Cap-D2*, which encodes a subunit of condensin I, and *Cap-H2*, which encodes a subunit of condensin II (Bauer et al., 2012; Hartl et al., 2008; Hirano, 2016). Expression of *Cap-H2* was unchanged in the absence of Mip120, whereas *Cap-D2* levels showed a moderate increase (Fig. S2).

## DISCUSSION

We have shown that the absence of Mip120 causes an arrest of *Drosophila* oogenesis during the transition between stages 7 and 8, with an accompanying abnormality of the germline nurse cell nuclei that includes persistent chromosome condensation, failure of chromosome disassembly, and failure of chromosome dispersion. This ovarian phenotype was observed with two independent mutant alleles of *mip120*, as was greatly reduced adult viability and longevity. However, we did not observe the previously described eye abnormalities, which may have been due either to another mutation or to interaction with a different genetic background. Mosaic analysis with a new loss-of-function allele of *mip120* demonstrated that the ovarian phenotype is caused by a lack of Mip120 within these germline cells, not the adjacent somatic follicle cells. Aberrations of nurse cell chromosomal disassembly can be caused by some mutant alleles of *ovarian tumor*, *suppressor of Hairy wing*, *RNA-binding protein 2/fs(2)B*, *cup*, *string of pearls*, *morula*, *Cap-H2*, and *rhino* (Cramton and Laski, 1994; Hartl et al., 2008; Keyes and Spradling, 1997; King et al., 1981; Klug et al., 1968; Reed and Orr-Weaver, 1997; Volpe et al., 2001). We have shown that ectopic expression of the female-specific ovarian HP1-related Rhino protein cannot rescue the *mip120* null mutant nurse cell phenotype. In addition, we did not observe any significant decrease in *Cap-H2* gene expression in the absence of Mip120. The relationship of *mip120* to the other genes that are known to affect nurse cell chromosome disassembly remains to be explored.

*Drosophila* RBF1 has been reported to interact directly with the Cap-D3 subunit of condensin II (Longworth et al., 2008). Therefore, it is also possible that Mip120 plays a role in directly regulating this condensin via the DREAM complex that also contains RBF1. Another possibility is that increased expression of *polo*, which encodes a protein kinase that phosphorylates and interacts with condensins and cohesins, may be responsible for inhibiting chromosome decondensation and disassembly in the absence of Mip120 (Alexandru et al., 2001; St-Pierre et al., 2009; Sumara et al., 2002). Similarly, increased expression of *aurA* and *aurB*, which encode protein kinases that regulate chromosome condensation and sister chromatid cohesion, may be partially responsible for the *mip120* mutant nurse cell phenotype (Crosio et al., 2002; Dai et al., 2006; Giet and Glover, 2001; Hsu et al., 2000; Resnick et al., 2006).

Mip120 is a sequence-specific DNA-binding protein that is a component of the MuvB core (Beall et al., 2002; Marceau et al., 2016; Schmit et al., 2009) (Fig. 1). In addition to this core, the DREAM complex contains the sequence-specific dE2F2-dDP DNA-binding heterodimer, whereas the MCC contains the sequence-specific Myb DNA-binding protein, and the large holocomplex contains all of these proteins (Korenjak et al., 2004; Lewis et al., 2004; Lipsick, 2004; Sadasivam and DeCaprio, 2013; van den Heuvel and Dyson, 2008). Previous studies have shown that dE2F2 is not essential for adult viability or female fertility (Cayirlioglu et al., 2001; Frolov et al., 2001). Surprisingly, the highly conserved DNA-binding domain of dMyb is not essential for adult viability but is required for fertility (Andrejka et al., 2011; Wen et al., 2008). We therefore wondered if the DNA-binding domains of dE2F2-dDP or dMyb might be able to compensate for the absence of the Mip120 DNA-binding domain. However, the failure of a mutant of Mip120 lacking its DNA-binding domain to rescue nurse cell nuclear morphology, chromosome disassembly, and arrest of oogenesis during the transition between stages 7 and 8 argues that the Mip120 DNA-binding domain is essential for female fertility.

The less conserved amino terminus of Mip120 was also required for rescue of these mutant phenotypes. In addition, the amino terminus was sufficient for nuclear localization. In this regard, previous studies have shown that the human LIN54 protein has at least two nuclear localization sequences, one in the amino terminus and one adjacent to the CXC domain (Matsuo et al., 2012). The PSORT algorithm predicts the presence of three potential nuclear localization sequences in *Drosophila* Mip120, two in the amino terminus (520-PRKHRLT and 550-PEAKKPR) and one directly adjacent to the CXC domain (737-RRKH) (Horton et al., 2007). However, a protein fragment containing the latter motif and the entire CXC domain (727-860) was not sufficient for nuclear localization. Neither was the HCH domain alone localized within the nucleus, although both of these mCherry fusion proteins are below the nominal 60 kDa limit for exclusion by the nuclear pore complex (43 kDa and 37 kDa, respectively) (Table S2) (Nigg, 1997). A larger carboxy-terminal fragment of *Drosophila* Mip120 containing both the CXC and HCH domains was sufficient for nuclear localization. However, this fragment was not capable of rescuing the mutant ovarian phenotypes. These results imply that the amino terminus of Mip120 has an essential but currently unknown function. Possibilities that remain to be explored include direct interaction with the L(3)MBT chromatin-binding tumor suppressor protein and with insulator-binding proteins (Blanchard et al., 2014; Bohla et al., 2014; Korenjak et al., 2014).

The expression of *bgcn* in the ovary is normally restricted to a small number of germline cells near the anterior tip of the germarium (Ohlstein et al., 2000). Remarkably, the expression of *bgcn* is increased greater than tenfold in Mip120-deficient ovaries. However, the expression of *bam* and *tut*, which encode proteins that can form a complex with Bgcn protein, was not increased (Chen et al., 2014; Li et al., 2009). Interestingly, the germline stem cells directly adjacent to the terminal filament within the germarium normally express *bgcn*, but not *bam* (Chen et al., 2014). Therefore, it appears that the absence of Mip120 results in a pattern of gene expression that at least in part resembles that of germline stem cells. The failure of chromosome disassembly in the germline nurse cells in *mip120* mutant ovaries suggests that Mip120 may play a broader role in repressing earlier programs of oogenesis as differentiation proceeds. Interestingly, loss-of-function mutants of *lin-54* and other synMuvB genes in *C. elegans* cause the depression of germline gene expression in somatic cells (Petrella et al., 2011; Tabuchi et al., 2014; Wang et al., 2005). Together these results suggest that the repression of germline gene expression by LIN54 and Mip120 family of proteins may have arisen prior to the evolutionary divergence of insects and nematode worms.

## MATERIALS AND METHODS

### Fly strains and genetics

To generate *mip120* null adult females of the genotype *w^1118^*; *mip120*^67-9a-9^/*Df(2R)BSC274*, we mated flies from two previously described strains: *w^1118^*; *mip120*^67-9a-9^/CyO and *w^1118^; Df(2R)BSC274*/CyO.

In some experiments a derivative of the CyO balancer that also contains a *P{w^+mC^ act::GFP =pActGFP}* transgene was used to permit unequivocal identification of balancer-free progeny by live fluorescence microscopy (Bloomington Drosophila Stock Center).

In other experiments transgenes on the third chromosome encoding fluorescent proteins were introduced as indicated into flies of the *w^1118^; mip120^67-9a-9^/Df(2R)BSC274* genotype or *mip120^WT^* controls. These transgenes included *P{RFP-HP1}3* (Wen et al., 2008) and *P{w[+mC]=GFP-cid.H}* (Schuh et al., 2007), or *P{GAL4::VP16-nos.UTR}* (Van Doren et al., 1998) and *P{UAS-GFP-Rhino*} (Klattenhoff et al., 2009). The *mip120^WT^* control flies used in this study were *w^1118^.* All flies were maintained at 25°C on standard cornmeal-dextrose-yeast medium unless otherwise indicated.

To test for rescue of the *mip120* null phenotype by various *mip120* transgenes described below, we crossed *w; mip120*^67-9a-9^/CyO; *P{mip120* transgene*}/MKRS* males to *w*; *Df(2R)BSC274*/CyO females. Female progeny of the genotype *w; mip120*^67-9a-9^/*Df(2R)BSC274; P{mip120* transgene}/+ were identified by the absence of the dominant *Curly* and *Stubble* phenotypes and were used to score for rescue of ovarian phenotypes. Female progeny of the genotype *w*; *mip120*^67-9a-9^/*Df(2R)BSC274; MKRS*/+ were identified by the absence of the *Curly* phenotype and the presence of the *Stubble* phenotype and were used as negative controls for the rescue experiments.

*P{mip120 full-length}*, *P{mip120 N-terminal}*, *P{mip120 C-terminal}*, *P{mip120 CXC}*, and *P{mip120 HCH}* plasmids described below were injected into *w^1118^* embryos using standard methods (BestGene, Inc., Chino Hills, CA, USA) to generate transgenic fly lines, which were then maintained as balanced stocks.

A new mutant allele of *mip120* that did not disrupt the essential *EfTuM* gene nested within the first intron of *mip120* was identified by screening publicly available lethal or semi-lethal transposon insertions in the region for rescue by the *P{mip120 full-length}* transgene. As described in the Results section, such an allele was present in a line obtained from the Kyoto Stock Center that had originally been generated in a large-scale mosaic screen (Schuldiner et al., 2008): y* w*; P{neoFRT}40A P{FRT(w^hs^)}G13 cn^1^ PBac{SAstopDsRed}LL07629 bw^1^/CyO, S* bw^1^.

Additional stocks from the BDSC were then used then used to generate flies with the following genotype for mosaic analysis: P{ry[+t7.2]=hsFLP}1, y[1] w[1118]/y* w*; *P{neoFRT}40A P{FRT(w^hs^)}G13 cn^1^ PBac{SAstopDsRed}LL07629 bw^1^/P{w[+mW.hs]=FRT(w[hs])}G13 P{w[+mC]=Ubi-GFP.nls}2R1 P{Ubi-GFP.nls}2R2*.

Somatic clones were induced by daily heat-shocks of larvae and pupae for one hour at 37°C. Newly eclosed adult females were mated with males, then maintained on standard food supplemented with yeast paste for various times prior to dissection of ovaries.

### Plasmid constructs

*P{mip120 full-length}*, *P{mip120 N-terminal}*, *P{mip120 C-terminal}*, *P{mip120 CXC}*, and *P{mip120 HCH}* plasmids encode full-length or fragments of Mip120 fused at their N-termini to mCherry and expressed under the control of the native genomic *mip120* promoter.

The native genomic *mip120* promoter was PCR amplified from the pw8_Mip120 plasmid using Platinum *Pfx* DNA Polymerase (Invitrogen), then cloned into the pCR4.0_TopoTA plasmid (Invitrogen). The following primers were used: GCATGCGTTACTCAGTGGCCAATT (5’ end) and GAATTCGCTGGGTGTGTATTGTGTAT (3'end).

An *SphI* site (underlined) was added to the 5’ end and an *EcoRI* site (underlined) was added to the 3’ end of the genomic *mip120* promoter. The genomic promoter of *mip120* was then swapped with the *UAS Hsp70* promoter in *UAS mCherry-mip120* plasmids.

*UAS mCherry-mip120*, *UAS mCherry-mip120 N-terminal*, *UAS mCherry-C-terminal*, *UAS mCherry-CXC*, and *UAS mCherry-HCH* plasmids were constructed in a pUAST backbone with PCR-amplified cDNA fragments from *pGEX_Mip120* using Platinum *Pfx* DNA polymerase (Invitrogen) with the following primers: GGATCCCTTAAGATGGACACGAGTGGCG (*mip120* 5’ end), GAATTCGCGGCCGCCTAAGAAGAAGGCTTGGA (*mip120* 3’ end), GGATCCCTTAAGATGGACACGAGTGGCG (*mip120 N-terminal* 5’ end), GAATTCGCGGCCGCCTAGGAAGCTGCCTTCTGT (*mip120 N-terminal* 3’ end), GGATCCCTTAAGCAGCCAGTTCAGAAACTA (*mip120 C-terminal* 5’ end), GAATTCGCGGCCGCCTAAGAAGAAGGCTTGGA (*mip120 C-terminal* 3’ end), GGATCCCTTAAGAAACCTCCAGCAACCGCG (*mip120 CXC* 5’ end), GAATTCGCGGCCGCCTATCCGTCCAGAGAGT (*mip120 CXC* 3’ end), GGATCCCTTAAGGAGGGTCAGAAAAAGGAC (*mip120 HCH* 5’ end), and GAATTCGCGGCCGCCTAAGAAGAAGGCTTGGA (*mip120 HCH 3’* end)*.*

*BamHI* and *AflII* restriction enzyme sites (underlined) were added to the 5’ end and *NotI* and *EcoRI* restriction sites (underlined) were added to the 3’ end of each amplified *mip120* cDNA fragment. These *mip120* fragments were first cloned into the pCR4.0_TopoTA plasmid (Invitrogen). The *mip120* fragments were then cloned into the intermediate plasmid vector *pSP72* using the *BamHI* and *EcoRI* sites.

The open reading frame encoding the *mCherry* fluorescent protein was amplified using the following primers: CCCTTAAGATGGTGAGCAAGGGCGAGG (5’ end) and CCCTTAAGCTTGTACAGCTCGTCCATGC (3’ end).

The *mCherry* ORF was then cloned in-frame into the *AflII* site (underlined) 5’ of each of the *mip120* ORFs. Each *mCherry-mip120* fragment was then cloned into the *BglII* and *NotI* sites of the UAST plasmid using the *BamHI* restriction enzyme site 5’ of *mCherry*-*mip120* and *NotI* restriction enzyme site 3’ of *mCherry-mip120.*

The primer pairs used for RT-qPCR are shown in Table S3.

### Egg-laying assay and analysis of ovary morphology

All flies were kept in a 25°C incubator. For the egg-laying assays, ten to 25 female virgins, which were 2-3 days old, were mated with ten to 25 *Ore-R* males. Flies were transferred every day to a fresh vial of food with yeast and yeast paste for three days. On the evening of the fourth day, flies were put in egg-collection chambers with 35×10 mm grape juice/agar plates (Fly Stuff/Genesee Scientific, San Diego, CA, USA) supplemented with yeast paste. Light streaks on the grape juice/agar plates were made to facilitate egg laying. Females were allowed to lay eggs on the plates in the dark for 13-15 h. The number of eggs on the plates was counted. A subset was followed for hatching into larvae after 24 h. Hatch rate was determined by the number of empty, clear eggshells divided by the total number of eggs. The female flies were then dissected in 1X PBS. The morphology of ovaries was observed under a Leica stereo microscope.

### Immunofluorescence

Before any ovary dissections, females were mated with males and kept on fresh vials of food with yeast and yeast paste for 2 days. Ovaries were dissected in PBS with 0.1% Triton-X 100 (PBST), fixed in 4% formaldehyde in PBS for 25 min, and washed 3×5 min in PBST.

For antibody staining of adult ovaries, samples were incubated in PBS with 3% Triton-X 100 for 1 h, washed 3×10 min in PBST, and then incubated with primary antibody diluted in PBST with 10% normal goat serum overnight at 4°C. All of the subsequent steps were done with the samples covered because of the light-sensitive nature of the secondary antibody fluorophores and the TO-PRO-3 nucleic acid dye (Molecular Probes). The samples were incubated in secondary antibody diluted in PBST with 10% normal goat serum (NGS) for 1 h and washed 3×10 min in PBST. After antibody staining, when necessary, samples were incubated with nucleic acid dye TO-PRO-3 diluted 1:1000 in PBST for 15 min and washed 3×5 min in PBST. Unless otherwise noted, all of the steps were conducted with gentle rotation at room temperature. Samples were mounted in Vectashield (Vector Labs) and immunofluorescence images were taken with a laser scanning confocal microscope. Primary antibodies used were mouse ADL67.10 anti-lamin (Developmental Studies Hybridoma Bank), rabbit anti-mCherry (Abcam), and rabbit anti-Mip120 (Lipsick laboratory). Rabbit anti-mCherry was pre-absorbed with fixed and dechorionated *Drosophila w^1118^* embryos before use in immunostaining. Alexa Fluor-conjugated goat secondary antibodies and phalloidin were used at 1:500 (Molecular Probes).

For live-imaging of fluorescent proteins encoded by transgenes, ovaries were dissected in PBS, transferred to halocarbon oil 27 (Sigma Aldrich, CAS 9002-83-9) on a glass slide, coverslipped, and imaged immediately with a laser scanning confocal microscope.
